# Supplementary material for: Mindfulness versus Physical Exercise: Effects of Two Recovery Strategies on Mental Health, Stress and Immunoglobulin A during Lunch Breaks. A Randomized Controlled Trial
Source: Int J Environ Res Public Health. 2020 Apr 20;17(8):2839. doi: 10.3390/ijerph17082839 (PMC7215846; doi:10.3390/ijerph17082839)
Supplement: Supplementary file 1 [file ijerph-17-02839-s001.pdf]

Table S1. Results of the MLM *protocol analysis* for the main effects - group, time and interaction factors.

Variable	Interaction			Time			Group		
	F	p	ω^2	F	p	ω^2	F	p	ω^2
**GHQ**									
Coping	1.33	.25	.03	10.64	<.01*	.29##	3.57	.03*	.07#
Self-esteem	1.11	.36	.01	8.10	<.01*	.23##	1.04	.36	.01
Stress	1.06	.39	.01	7.17	<.01*	.20##	1.09	.34	.01
Total	1.53	.17	.04	14.77	<.01*	.36##	.85	.43	<.01
**PSQ**									
Harassment-SA	3.63	<.01*	.18##	6.35	<.01*	.18##	1.31	.27	.01
Overload	2.24	.04*	.09#	7.57	<.01*	.21##	.75	.48	<.01
Irritab.-fatigue	2.15	.05*	.09#	7.45	<.01*	.21##	1.64	.20	.02
Energy-joy	.51	.80	<.01	.94	.42	<.01	1.65	.20	.02
Fear-anxiety	1.37	.23	.03	6.78	<.01*	.20##	.28	.76	<.01
Self-realisation	1.09	.37	.01	5.20	<.01*	.15##	1.56	.22	.01
Total	2.45	.03*	.11#	7.90	<.01*	.22##	1.95	.15	.03
**IgA**	.77	.55	<.01	.88	.42	<.01	.98	.38	<.01

* $p < 0.05$. # ω^2. Moderate effect size. ## ω^2. High effect size.

Table S2. Means and standard deviations for the MF, PE and CG, at different time points, in those dependent variables that have been significant in the analysis of the main effects.

	Time			
**Variable / Group**	M (SD)	M (SD)	M (SD)	M (SD)
**Harassment-SA**	**Pre**	**Post**	**F.up 1**	**F.up 2**
MM	.48 (.20)	.42 (.16)	.39 (.17)	.42 (.24)
PE	.47 (.15)	.26 (.17)	.41 (.13)	.36 (.18)
CG	.45 (.15)	.44 (.17)	.44 (.18)	.42 (.20)
**Overload**				
MM	.76 (.19)	.62 (.15)	.66 (.18)	.65 (.20
PE	.76 (.13)	.59 (.13)	.63 (.10)	.71 (.16)
CG	.73 (.15)	.71 (.20)	.70 (.14)	.70 (.17)
**Irritab.-fatigue**				
MM	.65 (.21)	.59 (.17)	.53 (.24)	.49 (.18)
PE	.62 (.14)	.45 (.18)	.49 (.20)	.48 (.15)
CG	.60 (.14)	.58 (.19)	.61 (.17)	.55 (.17)
**Fear-anxiety**				
MM	.53 (.26)	.44 (.24)	.39 (.29)	.36 (.23)
PE	.47 (.18)	.31 (.25)	.40 (.23)	.35 (.17)
CG	.47 (.23)	.44 (.21)	.36 (.22)	.34 (.21)
**Self-realisation**				
MM	.57 (.23)	.54 (.18)	.51 (.19)	.47 (.19)

PE	.56 (.15)	.40 (.22)	.44 (.19)	.41 (.19)
CG	.53 (.20)	.50 (.21)	.53 (.26)	.49 (.23)
**Total PSQ**				
MM	.63 (.18)	.56 (.13)	.53 (.17)	.52 (.17)
PE	.61 (.11)	.43 (.16)	.50 (.15)	.49 (.14)
CG	.59 (.12)	.57 (.17)	.58 (.16)	.55 (.17)
**Coping**				
MM	6.96 (3.32)	4.26 (1.63)	4.35 (2.29)	5.79 (2.66)
PE	6.50 (1.70)	5.06 (1.39)	4.89 (2.08)	6.61 (3.73)
CG	7.40 (2.86)	6.39 (2.08)	5.79 (1.87)	6.05 (2.27)
**Self-esteem**				
MM	5.07 (3.91)	3.22 (3.49)	2.52 (2.69)	3.16 (2.73)
PE	3.63 (2.58)	1.63 (1.31)	2.00 (1.60)	2.89 (2.49)
CG	3.67 (2.93)	2.87 (2.14)	2.32 (1.95)	2.63 (2.01)
**Stress**				
MM	5.11 (2.04)	3.87 (1.91)	3.61 (1.70)	3.05 (2.22)
PE	4.77 (1.36)	2.88 (1.45)	3.42 (1.35)	4.11 (1.91)
CG	4.43 (1.77)	4.09 (1.31)	4.05 (1.43)	4.21 (1.51)
**Total GHQ**				
MM	17.14 (7.20)	11.35 (5.18)	10.48 (4.81)	12.89 (6.66)
PE	14.90 (4.71)	9.56 (2.78)	10.32 (4.22)	13.61 (6.52)
CG	15.50 (6.32)	13.35 (4.96)	12.16 (4.50)	12.89 (4.86)

Table S3. Comparisons of simple effects - time factor PSQ.

Variable	Group	Comparisons	M_{diff}	S.E.	p	C.I. 95%	
						LLCI	ULCI
**Total (PSQ)**	Mindfulness	Pre-Post-test	.09	.03	.03*	.01	.18
		Pre-Fup 1	.09	.03	.06	.00	.17
		Pre-Fup 2	.10	.03	.03*	.01	.19
	Physical exercise	Pre-Post-test	.10	.03	.03*	.01	.20
		Pre-Fup 1	.07	.03	.45	-.02	.17
		Pre-Fup 2	.08	.03	.44	-.02	.18
**Harassment**	Mindfulness	Pre-Post-test	.14	.04	.04*	.00	.28
		Pre-Fup 1	.04	.04	.39	-.05	.12
		Pre-Fup 2	.06	.04	.19	-.03	.15
	Physical exercise	Pre-Post-test	.18	.05	.01*	.03	.33
		Pre-Fup 1	.12	.05	.66	-.05	.28

		Pre-Fup 2	.12	.05	.48	-.04	.28
**Overload**	Mindfulness	Pre-Post-test	.15	.03	.00*	.05	.25
		Pre-Fup 1	.09	.03	.08	.00	.19
		Pre-Fup 2	.10	.04	.22	-.02	.22
	Physical exercise	Pre-Post-test	.16	.03	.00*	.05	.27
		Pre-Fup 1	.13	.03	.00*	.03	.24
		Pre-Fup 2	.10	.04	.22	-.02	.22
**Irritab.-Fatigue**	Mindfulness	Pre-Post-test	.11	.03	.02*	.01	.21
		Pre-Fup 1	.10	.03	.01*	.02	.19
		Pre-Fup 2	.15	.03	.00*	.05	.26
	Physical exercise	Pre-Post-test	.15	.03	.00*	.04	.26
		Pre-Fup 1	.12	.03	.01*	.02	.21
		Pre-Fup 2	.14	.03	.00*	.03	.25
**Fear-anxiet**	Mindfulness	Pre-Post-test	.14	.04	.04*	.00	.28
		Pre-Fup 1	.17	.05	.02*	.02	.32
		Pre-Fup 2	.17	.04	.01*	.03	.32
	Physical exercise	Pre-Post-test	.18	.05	.01*	.03	.33
		Pre-Fup 1	.12	.05	.66	-.05	.28
		Pre-Fup 2	.12	.05	.48	-.04	.28
**Self-realization**	Mindfulness	Pre-Post-test	.13	.05	.01*	.03	.22
		Pre-Fup 1	.04	.04	.39	-.05	.12
		Pre-Fup 2	.06	.04	.19	-.03	.15
	Physical exercise	Pre-Post-test	.13	.05	.02*	.03	.23
		Pre-Fup 1	.09	.05	.06	.00	.18
		Pre-Fup 2	.07	.05	.15	-.03	.17

* $p < .05$.

Table S4. Correlations between the amount of final practice and the degree of recovery (pretest - posttest change scores).

**Variable**	**Group**			
	**Mindfulness**		**Physical Exercise**	
	[1]r_{xy}	p	[1]r_{xy}	p
PSQ	.29	.16	.26	.25
GHQ	.34	.09	.19	.40
IgA	.12	.57	-.19	.41

[1.] Pearson's correlation coefficient.

$p < .05$
